# Supplementary material for: Testing for Hepatitis B and C virus and HIV in mental healthcare settings in England between 2015–2021
Source: PLOS Ment Health. 2024 Jul 10;1(2):e0000011. doi: 10.1371/journal.pmen.0000011 (PMC12798291; doi:10.1371/journal.pmen.0000011)
Supplement: Attachment — Submitted filename: Mental health - response to reviewers.docx [file pmen.0000011.s001.docx]

Dear Dr. Kizito Omona,

We would like to thank the editors and reviewers for their comments on our manuscript. We have addressed the changes suggested to the title, expanded the sub-headings in the results, and expanded the discussion on prevalence rates and ethnicity in the discussion. Please find below a more detailed to response to the suggestions.

Matthew Hibbert

On behalf of all co-authors

Revise your title to "Testing for Hepatitis B and C virus and HIV in mental healthcare settings in England between 2015-2021". The other words in the title look redundant and make your title not so easy to understand.

Thank you for this suggestion. The title has now been changed as suggested.

Add appropriate and complete sub-sections headings for your results. The present sub-section headings are fairly incomplete.

Thank you for this. The subheadings in the results have now been expanded upon.

Reviewer #1: In the discussion I missed presenting and discussing the possible reasons that may have interfered with the increased prevalence rates (HBV, HCV and HIV) in people with serious mental illnesses. Just like this difference in race profile.

Thank you for this. We have rearranged the discussion so that the section on prevalence rates is presented, then differences in gender and sexuality, and then ethnicity. We added the following text to explain the increased prevalence of BBVs among people with mental illness:

“This is because behaviours that are associated with potential exposure to HBV, HCV, and HIV have been observed at an increased prevalence among people with severe mental illness and mental illness generally compared to the general population (7-12).” (tracked changes manuscript: page 11, lines 300-303).

We have also added the following text to discuss ethnicity further:

“The association between White ethnicity and a positive HCV result is similar to what is seen nationally, due to the majority of people diagnosed with HCV and seen for HCV care are White British people who inject or have previously injected drugs (21, 28).” (tracked changes manuscript: page 11, lines 324-326).

“Therefore, it appears as though whilst there may be a higher prevalence of BBVs among people with mental illness, the variations in test positivity across different ethnicities is broadly representative of those diagnosed nationally.” (tracked changes manuscript: page 11, lines 328-330).

Reviewer #2: This study determines the Testing for Hepatitis B and C virus and HIV in mental healthcare settings and 2 engagement in care in England between 2015-2021. The study is well written and publishing it will open the eyes of other readers to concentrate on patients in mental health facilities and those suffering from mental health.
The authors should read through to correct minor mistakes.

Thank you for this. We have reread the manuscript and corrected minor typos.
